# The Effects of Low-Dose Bisphenol A and Bisphenol F on Neural Differentiation of a Fetal Brain-Derived Neural Progenitor Cell Line

**DOI:** 10.3389/fendo.2018.00024

**Published:** 2018-02-09

**Authors:** Yuki Fujiwara, Wataru Miyazaki, Noriyuki Koibuchi, Takahiko Katoh

**Affiliations:** ^1^Department of Public Health, Faculty of Life Sciences, Kumamoto University, Kumamoto, Japan; ^2^Department of Integrative Physiology, Graduate School of Medicine, Gunma University, Maebashi, Japan

**Keywords:** neural differentiation, bisphenol A, neuron, estrogen, environmental chemicals, brain development, bisphenol F

## Abstract

Environmental chemicals are known to disrupt the endocrine system in humans and to have adverse effects on several organs including the developing brain. Recent studies indicate that exposure to environmental chemicals during gestation can interfere with neuronal differentiation, subsequently affecting normal brain development in newborns. Xenoestrogen, bisphenol A (BPA), which is widely used in plastic products, is one such chemical. Adverse effects of exposure to BPA during pre- and postnatal periods include the disruption of brain function. However, the effect of BPA on neural differentiation remains unclear. In this study, we explored the effects of BPA or bisphenol F (BPF), an alternative compound for BPA, on neural differentiation using ReNcell, a human fetus-derived neural progenitor cell line. Maintenance in growth factor-free medium initiated the differentiation of ReNcell to neuronal cells including neurons, astrocytes, and oligodendrocytes. We exposed the cells to BPA or BPF for 3 days from the period of initiation and performed real-time PCR for neural markers such as β III-tubulin and glial fibrillary acidic protein (GFAP), and Olig2. The β III-tubulin mRNA level decreased in response to BPA, but not BPF, exposure. We also observed that the number of β III-tubulin-positive cells in the BPA-exposed group was less than that of the control group. On the other hand, there were no changes in the MAP2 mRNA level. These results indicate that BPA disrupts neural differentiation in human-derived neural progenitor cells, potentially disrupting brain development.

## Introduction

A variety of environmental chemicals are known to induce adverse effects in animals and humans including general toxicity (e.g., organ damage), carcinogenesis, mutagenesis, and reproductive toxicity. The risk assessment of these toxic substances is done according to OECD guidelines. Recently, several animal studies have shown that gestational exposure to low-dose toxicants, such as dioxin, may cause adverse effects including neurodevelopmental alterations without affecting the dams (1, 2). These reports suggested that the developing brain is vulnerable to exposure to environmental chemicals, even at low doses. Many studies have demonstrated neurodevelopmental and behavioral disorders attributable to gestational and/or lactational exposure to environmental chemicals. We have previously shown that during the maternal and postnatal periods, dioxin can cross the developing blood–brain barrier and cause learning deficits, emotional abnormalities, and changes in social behavior (3–5).

During brain development, neural cells, including neurons and glial cells, are differentiated from neural stem/progenitor cells (NPCs) (6). The differentiation process is precisely and tightly controlled by many factors, including hormones and receptors. Estrogen is an important factor for neural differentiation, protection, and function. Estrogen acts by binding to nuclear estrogen receptors (ERs). The differentiation of dopaminergic (DA) neurons in the ventral mesencephalon (VM) (7) is also regulated by the estrogen–ER pathway. Several environmental chemicals, such as bisphenol A (BPA), isoflavones, and phthalates, which are also called phytoestrogen or xenoestrogen, are known to disrupt such pathways because of their estrogenic actions (8, 9). Among these, the toxicity of BPA is considered one of problems as disruptors on estrogenic pathway.

Large volumes of BPA are used to make toys, plastic containers, and dental pastes (10–12). BPA is readily eluted from these products and delivered to the human body through foods and drinks. Consequently, BPA has been detected in the tissue including the brain of human fetuses, babies, and pregnant mothers (13). The distribution of BPA in both fetuses and babies reflects that BPAs are delivered through placenta and breast milk. In fact, BPA was detected in fetal cord blood, placental tissues, and breast milk (14, 15). Surprisingly, the metabolites of BPA can also cross the placenta, and such metabolites are deconjugated to BPA in fetuses (16). Transplacental and lactational exposure may cause different adverse effect. In addition, the differences of these adverse effects may depend on not only the timing of exposure to BPA in transplacental (fetal) or lactational but also sex in mice and rat (17). This hypothesis is evidenced further by a human epidemiological study, showing that gestational BPA exposure induced sex-dependent associations with aggressive behaviors in children and adolescents (18). Thus, we consider it important to investigate the effects of BPA on brain development, especially neural differentiation. Recent studies also showed that low-dose BPA exposure causes toxicological effects in the brain (19). BPA induces estrogen-like effects *via* ERs (20–22), disrupting sexual differentiation in the developing brain and changing sex-dependent behaviors by perinatal exposure. Past studies revealed sex-based behavioral differences following maternal exposure to BPA in learning and memory, novel exploration, and emotional behavior (23–25). BPA exposure increases locomotor activity and anxiety-like behavior (26, 27), decreases impulsive behavior, and changes social behaviors (24, 28). Although the precise mechanisms that cause these behavioral alterations are unclear, some studies have indicated that BPA exposure suppresses synapse formation (29), disrupts neural migration (30, 31), increases the number of glial cells (32, 33), and upregulates neural cytoskeletal proteins (32, 34). *In vitro* studies using cell lines showed the inhibition of dopamine release (35) and the augmentation of the microtubule-associated protein 2 (MAP2) mRNA expression. MAP2 is a cytoskeleton-related protein in neurons, and its expression is changed in the presence of neurodegenerative diseases such as schizophrenia (19, 20). These changes may cause abnormal brain development and behavioral alterations. BPA may exert adverse effects on normal brain development by disrupting neural differentiation, including DA neurons in the VM. However, the mechanisms of BPA action have not yet been fully clarified, especially in humans.

To avoid BPA exposure, several alternative substances are produced. Bisphenol F (BPF) is one such compound that is used in epoxy resins and coatings. Although the exposure levels of BPF in the environment, humans, and wild animals are lower than those of BPA, BPF may also affect human health, including brain development. However, to our knowledge, the effects of BPF on neural differentiation from NPCs in humans have not yet been reported.

In this study, to examine the effects of BPA and BPF exposure during neural differentiation, we used a human fetal VM-derived NPC cell line, ReNcell VM cell line. This cell line is appropriate to investigate the effects on the neural differentiation of human, because this was derived from a 10-week human VM brain tissue and established as an NPC cell line with immortalization. Moreover, as stated above, BPA and BPF may disrupt the differentiation through ERs because of the potencies of their estrogen-like effects. We then examined the changes in neuronal differentiation attributable to BPA or BPF exposure.

## Materials and Methods

All experiments in this study were performed under the restrictions in biosecurity and safety procedures of Gunma University.

### Chemicals

Bisphenol A (99% purity) and BPF (99% purity) were purchased from Sigma-Aldrich (MO, USA). BPA and BPF were dissolved in ethanol and DMSO, respectively. β-estradiol (E2), 98% pure and dissolved in ethanol (99.5% purity), was also obtained from Sigma-Aldrich.

### Cell Culture

ReNcell VM (Millipore, MA, USA) is an immortalized NPC line derived from the VM of a 10-week-old human fetal brain. The cells were cultured as previously described (36). The passage of all cells used in this investigation was lower than 31, because previous work has shown that these cells maintain a stable karyotype up to 45 passages. Briefly, ReNcell VM cells were expanded in an expansion medium (ReNcell NSC Maintenance Medium, Millipore, MA, USA), supplemented with 20 ng/ml of epidermal growth factor (Millipore, MA, USA) and 20 ng/ml of basic fibroblast growth factor (Millipore, MA, USA) on laminin-coated (Wako Pure Chemical Industries, Ltd., Osaka, Japan) 1.7-µg/cm^2^ TC-treated culture flasks at 37°C in a 5% CO_2_ humidifier incubator. The medium was renewed every 2 days during proliferation, and the cells were subcultured approximately every 5 days (90% confluence) by detaching them using Accutase (Millipore, MA, USA). After each passage, cell concentration and viability were determined by counting with a hemocytometer (Hausser Scientific, Horsham, England) using the trypan blue dye (Invitrogen, CA, USA) exclusion test. After this, the cells were again seeded at 5 × 10^4^ cells/ml in freshly laminin-coated flasks. Differentiation of the cells was accomplished by adding fresh differentiation medium (ReNcell NSC Maintenance Medium without growth factors) to confluent monolayers of cells. Unless otherwise indicated, cells were incubated for 3 days in a differentiation medium, and the medium was changed every 2 days.

At the onset of differentiation, the cells were exposed to BPA, BPF, or E2 and incubated for 3 days. Control cells were exposed to solvents following the same protocol. E2-exposed group was used for positive control, because BPA and BPF have the potency to induce estrogen-like effects through ERs.

### Cell Cytotoxicity Assay

Cell cytotoxicity was measured using the Cell Counting Kit-8 (CCK-8; Dojindo, Osaka, Japan) according to the manufacturer’s protocol. Briefly, ReNcell VM cells (1 × 10^5^ cells/well) were seeded onto laminin-coated TC-treated culture 96-well plates and cultured with an expansion medium at 37°C in a 5% CO_2_ humidifier incubator. After 24 h, cells were incubated with a BPA-containing expansion medium for another 24 h. At the end of the culturing period, we added 10 µl of CCK-8 reagent to the cells and incubated them for 4 h at 37°C.

A water-soluble tetrazolium salt (WST-8) containing a CCK-8 reagent reacts with dehydrogenase from living cells and turns into WST-8 formazan, an orange dye. The fluorescence intensity correlates with the number of living cells in the sample. After incubation with CCK-8 reagents for 4 h, we measured the absorbance of light at 450 nm with a Synergy HTX plate reader (Biotek Instruments, Inc., VT, USA).

### Quantitative Real-time PCR

Total RNA was isolated using the miTotal™ RNA Extraction Miniprep System (VIOGENE, New Taipei City, Taiwan), and 2.0 µg of total RNA was reverse-transcribed into cDNA using ReverTraAce qPCR RT Master Mix (TOYOBO, Osaka, Japan). Quantitative real-time PCR was performed with specific primers (Table 1) in the StepOne thermal cycler (Applied Biosystems/Life Technologies, CA, USA) using THUNDERBIRD SYBER qPCR Mix (TOYOBO Japan) for 40 cycles according to the following program: a denaturation step at 95°C for 30 s followed by an annealing/extension step at 95°C for 30 s. The data were analyzed using the delta–delta Ct method. GAPDH was used for normalization.

**Table 1:** Primer sequences.

Primer	Forward	Reverse
β III-tubulin	CATGGACGAGATGGAGTTCA	TTCGTACATCTCGCCCTCTT
MAP2	CAGAAGTTCAGGCCCACTCT	GGTTTTCCGCTTAACACAGG
GFAP	GAGATCGCCACCTACAGGAA	CAGGCTGGTTTCTCGAATCT
S100β	GGGAGACAAGCACAAGCTG	TCCACAACCTCCTGCTCTTT
Olig2	GACAAGCTAGGAGGCAGTGG	GGCTCTGTCATTTGCTTCTTG
Nestin	GACTTCCCTCAGCTTTCAGG	TCAGGACTGGGAGCAAAGAT
Dcx	TCCCCAACACCTCAGAAGAC	GCGTAGAGATGGGAGACTGC
NCAM1	GAACCAGCAAGGAAAATCCA	CAGGACGAAGATGACGATGA
MCT1	TTGGTTGGCTCAGCTCTGTA	CAGCATTCCACAATGGTCAC
ESR1	GATGAATCTGCAGGGAGAGG	TCCAGAGACTTCAGGGTGCT
ESR2	TCAGGCATGCGAGTAACAAG	TCCAGCAGCAGGTCATACAC
Rtp1	GGTTGGAAGCAGTACCTGGA	GGTCCAGGAACATGTGGAAG
GAPDH	GATCATCAGCAATGCCTCCT	TGAGTCCTTCCACGATACCA

### Immunocytochemistry

Following growth and differentiation on laminin-coated chamber slides, cells were washed with PBS and fixed with 3% paraformaldehyde solution in PBS for 30 min. After washing twice with PBS, cells were incubated with 0.1% triton X-100 solution and were blocked in PBS containing 10% BSA for 1 h at room temperature. Subsequently, cells were incubated with the following antibodies: β III-tubulin (Sigma-Aldrich, MO, USA) (1:500 dilution) and glial fibrillary acidic protein (GFAP) (Abcam, Cambridge, England) (1:500 dilution) overnight at room temperature. After washing with PBS, cells were incubated with the following secondary antibodies for 1 h in the dark at room temperature. Cells were then washed with PBS again, and the nuclei were counterstained with 1 µg/ml Hoechst 33342 (Sigma-Aldrich, MO, USA) for 10 min, in the dark. Stained cells were washed with PBS and were observed with a fluorescence microscope (EVOS fl: Thermo Fisher Scientific, MA, USA).

### Statistical Analysis

Experimental data were analyzed using GraphPad Prism (GraphPad Software, CA, USA). Results are represented as mean ± SEM. Statistical analysis was performed by one-way analysis of variance (ANOVA) followed by the Tukey test. In Figures 2 and 6B, Student’s *t*-test was used to compare two groups. For all comparisons, the results were considered to be significant if the *p*-value was <0.05.

## Results

### BPA Did Not Induce ReNcell VM Cell Death

**Figure 1:**
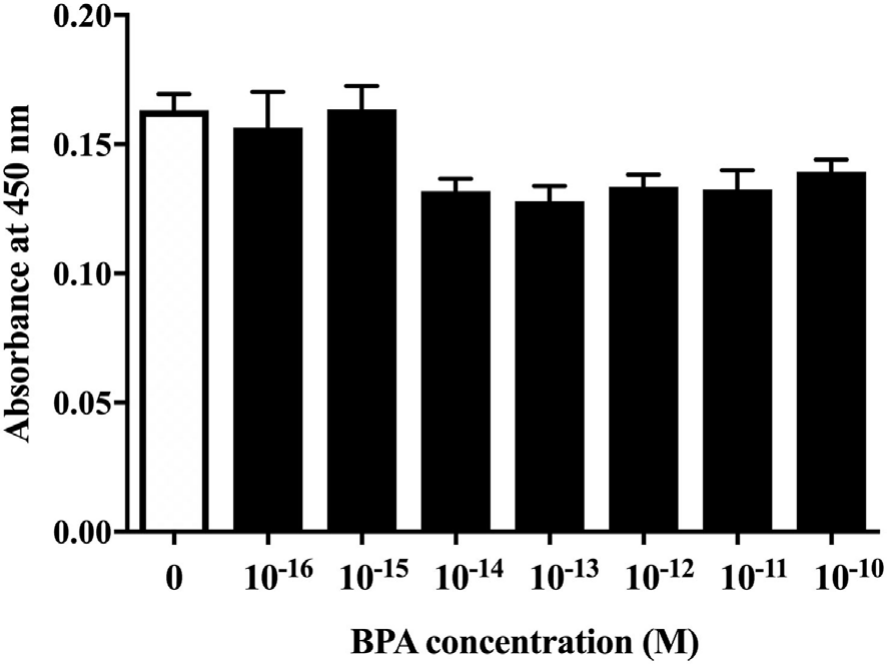
The cell viability of ReNcell ventral mesencephalon cells was assessed after a 24-h exposure to BPA or a control reagent. The cell viability assay was performed using a Cell Counting Kit-8 kit (see Materials and Methods section for details). Data are represented as mean ± SEM (*n* = 7/group).

First, to examine whether BPA exposure induced ReNcell VM cell death, we investigated the cytotoxicity of BPA using a CCK-8 (DOJINDO LABORATORIES, Kumamoto, Kyushu, Japan). The amount of formazan dye, measured as the absorbance of light at 450 nm, is proportional to the number of living cells. There was no difference in the absorbance, indicating that BPA exposure did not induce cell death at concentrations between 10^−16^ and 10^−10^ M (Figure 1).

### BPA Induced a Dose-Dependent Decrease in Both β III-Tubulin and S100 β mRNA Levels

**Figure 2:**
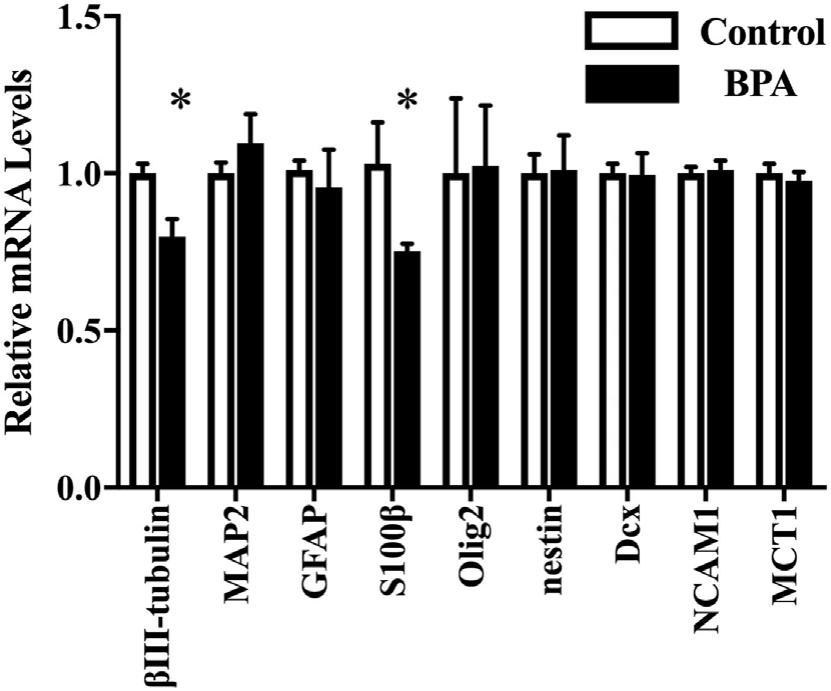
mRNA levels of several genes in differentiated-ventral mesencephalon (VM) cells. mRNA levels of β III-tubulin, glial fibrillary acidic protein (GFAP), Olig2, nestin, Dcx, NCAM1, and MCT in differentiated ReNcell VM cells after exposure to 10^−10^ M bisphenol A (BPA). Data are represented as mean ± SEM, *n* = 3. The control group values were defined as 1. *p < 0.05.

**Figure 3:**
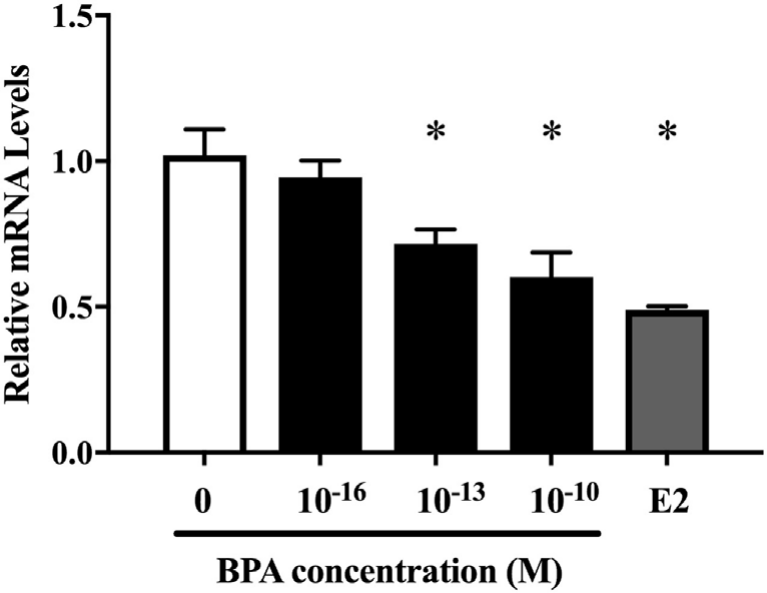
Dose-dependent differences in mRNA levels of β III-tubulin in differentiated ReNcell ventral mesencephalon cells following exposure to 10^−16^ and 10^−10^ M bisphenol A (BPA) and 10^−10^ M β-estradiol. Expression levels of β III-tubulin were analyzed by a real-time quantitative PCR assay. Data are represented as mean ± SEM, *n* = 3.

Next, we investigated the ratio of differentiated cell types using a real-time PCR method with various neural markers, such as βIII-tubulin for neurons during differentiation, MAP2 for mature neuron, GFAP and S100β for astrocytes, Olig2 for oligodendrocytes, and nestin, Dcx, NCAM 1, and MCT 1 for NPCs. The expression of both ESR1 (ERα) and ESR2 (ERβ) was also confirmed in ReNcell VM cells. We observed a decrease in β III-tubulin and S100β mRNAs induced by 10^−10^ M BPA treatment (Figure 2). The expression of other markers was not altered. ESR1 and ESR2 mRNA levels were not altered by the exposure to BPA (Figure S1 in Supplementary Material). Their expression was not altered by BPF or E2 treatment. To verify the dose dependency of the effect of BPA on β III-tubulin, cells were exposed to various concentrations of BPA. E2 (10^−10^ M) was used as a positive control. BPA at both 10^−13^ and 10^−10^ M significantly decreased β III-tubulin levels (Figure 3). Analysis by ANOVA showed a significant dose-dependent effect [*F*(4, 17) = 9.688; *p* = 0.0003]. E2 also decreased β III-tubulin levels.

### BPA Reduced the Number of β III-Tubulin-Positive Cells

**Figure 4:**
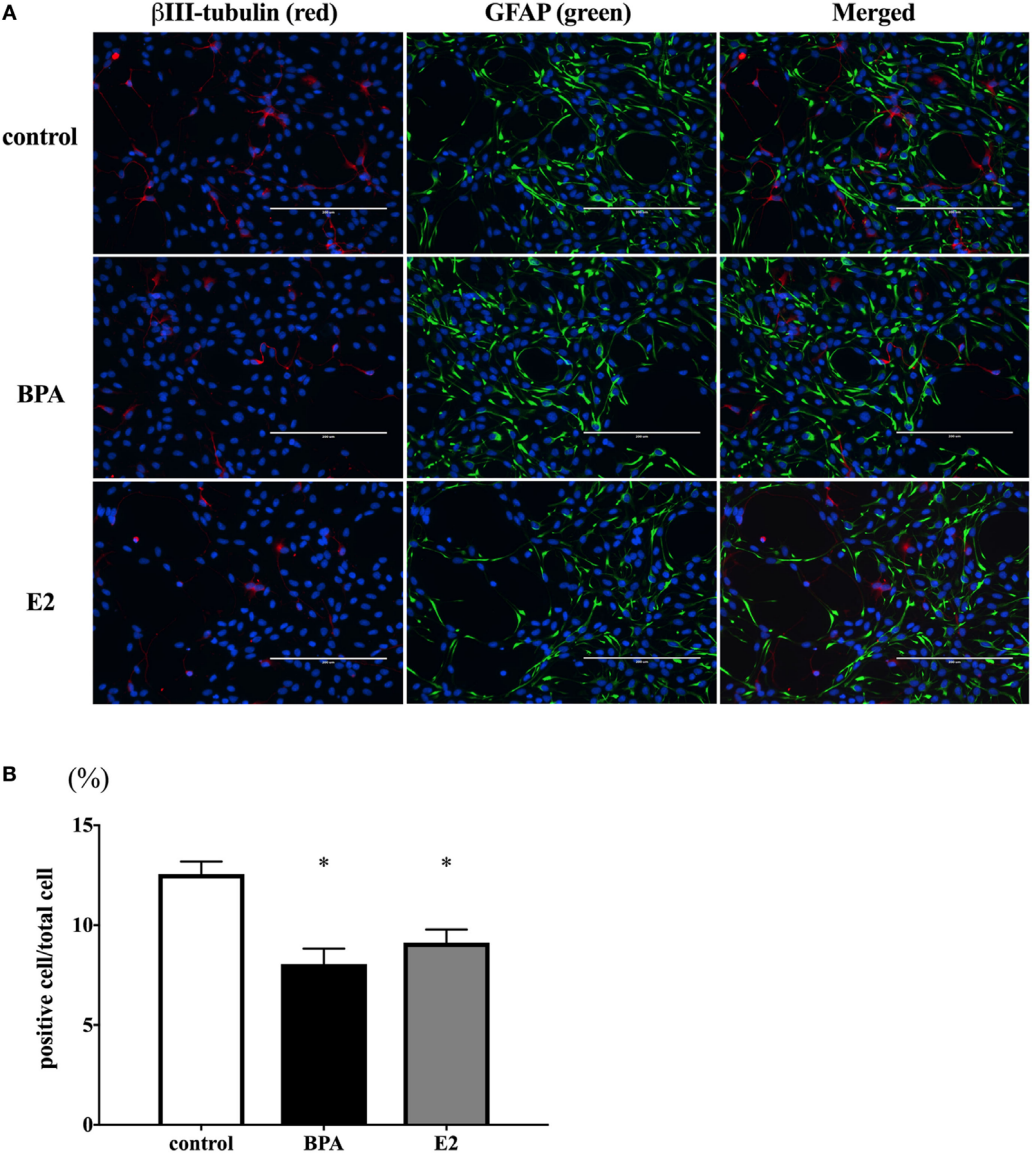
Number of β III-tubulin-positive cells. **(A)** ReNcell ventral mesencephalon cells treated with bisphenol A (BPA), β-estradiol (E2), or control were immune-stained with antibodies for β III-tubulin (red) and glial fibrillary acidic protein (GFAP) (green). Nuclei were identified by Hoechst 33342 staining. Panel **(B)** shows the ratio of β III-tubulin-positive cells per total cells. The number of β III-tubulin-positive cells was counted with four arbitrarily selected fields per well (scale bar: 200 µm). Data are represented as mean ± SEM, *n* = 47.

We hypothesized that the decrease of β III-tubulin mRNA levels by BPA may be due to the decrease of the number of differentiated neurons. To test this hypothesis, we performed immunofluorescence staining with both anti-β III-tubulin and anti-GFAP antibodies, along with Hoechst staining for nucleus. The number of β III-tubulin-positive cells was counted and expressed as a percentage of the total number of cells (the number of Hoechst-positive cells) (Figure 4A). We observed that the percentage of β III-tubulin-positive cells decreased significantly in the BPA-exposed group compared to that in controls (Figure 4B). In addition, E2 also decreased the percentage of β III-tubulin-positive cells.

### BPF Did Not Affect β III-Tubulin Levels

**Figure 5:**
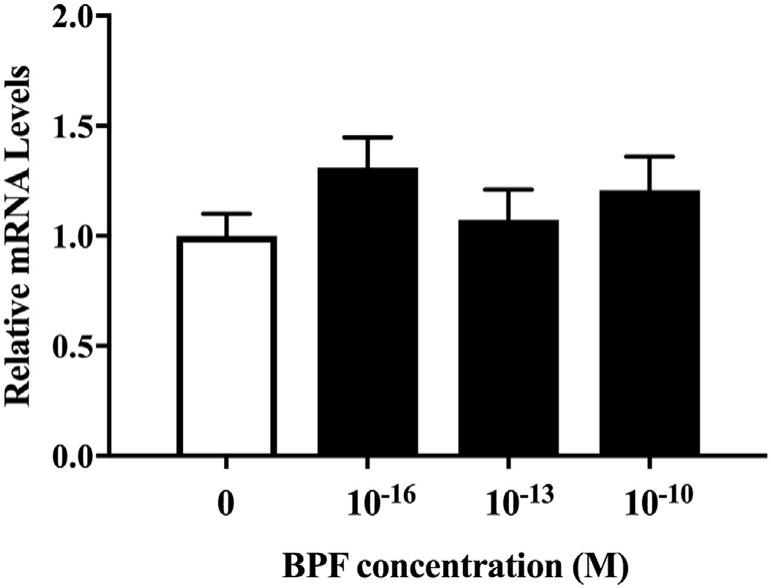
mRNA levels of β III-tubulin in differentiated ReNcell ventral mesencephalon cells exposed to bisphenol A. Cells were harvested after 3 days exposure to 10^−16^, 10^−13^, and 10^−10^ M bisphenol F (BPF) and extracted total RNA. Real-time quantitative PCR analyses were performed using a primer set for β III-tubulin. Data are represented as mean ± SEM, *n* = 3.

**Figure 6:**
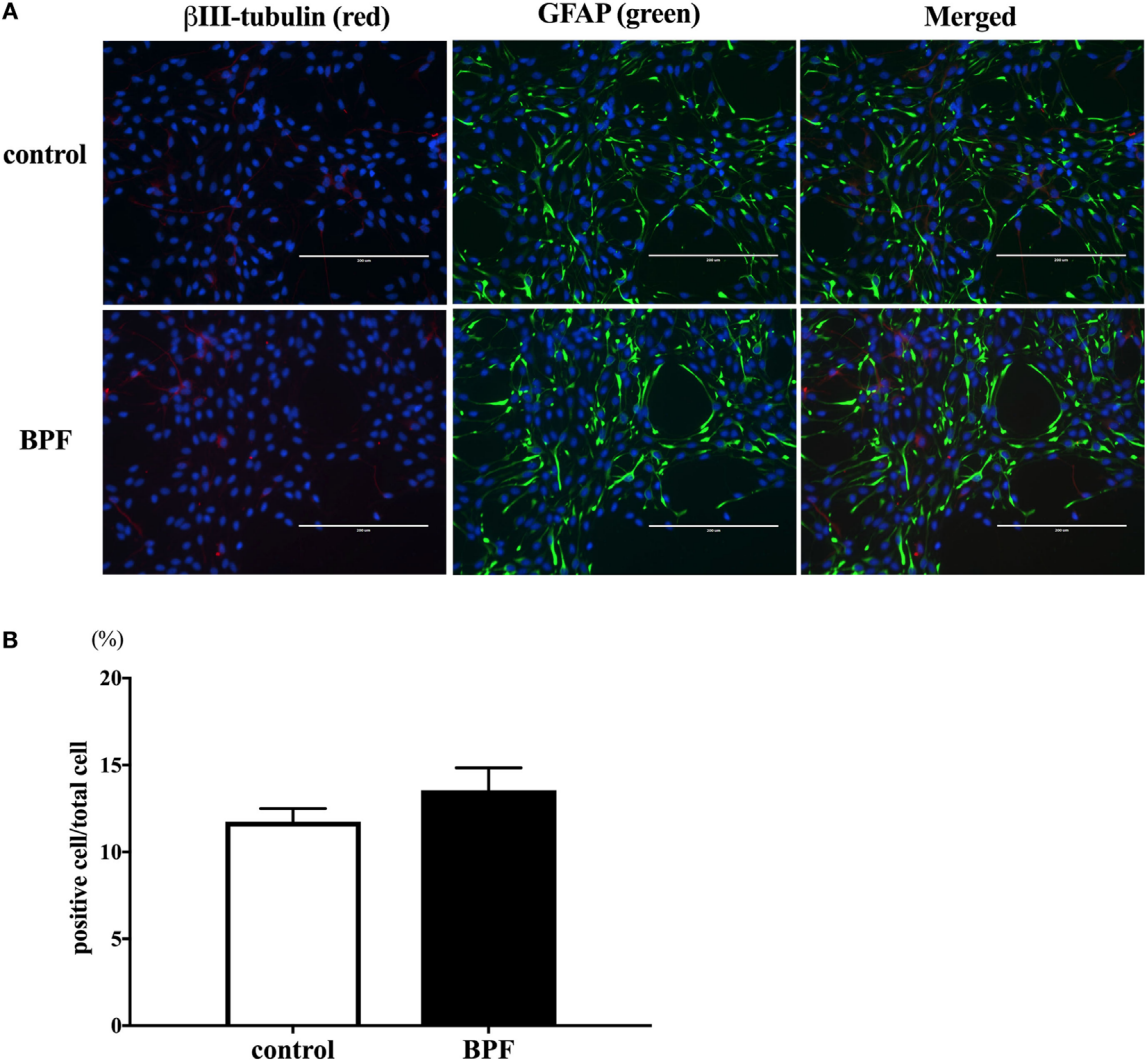
The effects of bisphenol F (BPF) on the number of β III-tubulin-positive cells. **(A)** We performed immunostaining of ReNcell ventral mesencephalon cells treated with BPF or control reagents with antibodies for β III-tubulin (red) and glial fibrillary acidic protein (GFAP) (green), and Hoechst 33342 solution was used for nuclei staining (scale bar: 200 µm). **(B)** The ratio of β III-tubulin-positive cells to total cells was measured. Data are represented as mean ± SEM, *n* = 3.

We also investigated whether BPF induced changes similar to BPA using RT-PCR and immunofluorescence staining for β III-tubulin. To compare the effects of BPF and BPA exposure, we used the same concentration of BPF (10^−10^ M) as was used for BPA. RT-PCR (Figure 5) and immunofluorescence staining (Figures 6A,B) showed no significant alteration in response to BPF exposure.

## Discussion

In this study, we investigated the effects of BPA exposure on neural differentiation from NPCs. Exposure to BPA for 3 days from the initiation of neural differentiation decreased both β III-tubulin and S100 β mRNAs without inducing cell death. The number of β III-tubulin-positive cells was decreased significantly following BPA exposure; by contrast, BPF, a substitute for BPA, did not alter β III-tubulin mRNA levels or the number of β III-tubulin-positive cells. Moreover, we observed similar changes in β III-tubulin mRNA and β III-tubulin-positive cells following E2 treatment. Together with previous studies showing that BPA may act as a “xenoestrogen,” these results suggest that BPA exposure affects neural differentiation through estrogen-like and/or ER-mediated pathways. In addition, BPF may be a useful substitute for BPA and may avoid the adverse effects of BPA on neural differentiation.

The effects of BPA on neural differentiation using primary cell culture methods using NPCs of the telencephalon or hippocampus have been reported. Using NPCs of the rat hippocampus, Agarwal et al. showed that BPA caused oxidative stress in mitochondria of the NPCs. Such mitochondrial dysfunction inhibited the proliferation and differentiation to β-III-tubulin-positive neuronal cells (37). We also showed the decrease of the number of β-III-tubulin-positive neuronal cells by BPA without alteration of the expression level of MAP2 mRNA. These results indicate that BPA accelerated the differentiation in the later stage from β-III-tubulin-positive neuronal cells to MAP2-positive neuron but not in the earlier stage from NPCs to β-III-tubulin-positive neuronal cells. On the other hand, there were no effects in oligodendrocytes differentiation in our study. Okada et al. reported that exposure to BPA increased the ratio of oligodendrocytes differentiated from NPCs of the rat telencephalon (38). However, other report showed that BPA reduced the differentiation to oligodendrocytes from NPCs of the rat hippocampus (39). Moreover, there are no reports showing the change in astrocyte differentiation by BPA. These results, including our study, suggest that BPA may disrupt neural differentiation from NPC to neuron, whereas further study may be required to confirm its effect on differentiation to oligodendrocyte or astrocyte.

Estrogen and its nuclear receptors, ERs, regulate brain development during differentiation, growth, and the acquisition of brain function (40, 41). Moreover, protection from several toxicants, cellular survival, and neuronal peptides synthesis are also induced by estrogen. In the neural differentiation process, E2 induces the expression of neurotrophic factors such as brain-derived neurotrophic factor, neural growth factor, and neurotropin-3. Such increases in neurotrophic factor expression may affect the proliferation of NPCs, the ratio of neurons to glial cells, and neurite outgrowth. BPA may also bind to ERs and exert agonistic and/or antagonistic activities in humans and animals, potentially causing neurotoxicity, reproductive toxicity, and immune toxicity. The binding affinity of BPA to ERs is 1/500–1/15,000 smaller than that of E2 (21, 42, 43) in humans and rats. Nevertheless, BPA can strongly activate ER-mediated transcription in transient transfection-based reporter gene assay (20, 42, 44, 45). In the present study, to examine whether the effect of BPA is induced through ER, it would be useful to examine the change in estrogen-responsive genes in differentiating human brain cells. However, such gene has not yet been identified. A previous report showed that the exposure to BPA or BPF induced the expression of estrogen-sensitive markers, cyp19a1b, in developing zebra fish brain (46). Unfortunately, this gene is not expressed in mammalian brain. Other candidate estrogen-responsive genes are Nlrp3 and Rtp1, which are expressed in adult mice cerebral cortex (47). Thus, we examined the change in these mRNA levels during differentiation. However, we could not observe any changes of the expression of Rtp1 even by the exposure to E2 (Figure S2 in Supplementary Material), and Nlrp3 was not expressed in the differentiating cells. These results indicate that the marker that we used may not be appropriate to examine the effect of E2 and BPA in differentiating cells. To further investigate the involvement of ER-mediated pathway on BPA effect during neural differentiation, additional study including the identification of estrogen-responsive genes in differentiating cells may be necessary. Nevertheless, by considering the strong BPA action on ER-mediated transcription, the possibility that the BPA action seen in the present study is exerted through ER cannot be excluded.

Although BPA can activate ER-mediated transcription, its effect on ER-mediated action in neural differentiation is controversial. This is partly because E2 plays different roles in this process. ERs are expressed and may be involved in the differentiation of DA neurons (48). However, while E2 may mediate the proliferation and survival of NPCs (49), long-term E2 exposure induces the suppression of cell proliferation in the dentate gyrus (50). Thus, even if BPA acts through ERs, its action may be affected by the dose, length, and period of exposure (39). Huang et al. reported that BPA suppressed DA neuron differentiation in human embryonic stem cells by suppressing E2-activated insulin-like growth factor pathways (51). In the VM region, Elsworth et al. reported that prenatal BPA exposure decreased the number of DA neurons in the fetal VM and spine synapses in the hippocampus (52). In the present study, both BPA and E2 suppressed neural differentiation. Further study is required to differentiate the effects of BPA on ER-mediated regulation of DA neuron differentiation.

We also observed a decrease in S100β mRNA induced by BPA. S100β is often used as an astrocyte marker because it is specifically synthesized and secreted from astrocytes. Unfortunately, we could not find a good antibody to stain S100β protein for immunocytochemistry. Thus, we could not count the number of S100β-positive cells. On the other hand, mRNA levels of GFAP, another astrocyte marker, were not altered by BPA. These results indicate that BPA may specifically affect the expression or the secretion of S100β without changing the number of astrocytes. However, although we were able to immunostain GFAP, we could not obtain a stable count of the number of astrocytes, because astrocytes have long processes and relatively small stomata, both of which were strongly GFAP-positive. Thus, in the present study, we were unable to fully examine the effect of BPA on astrocyte differentiation. Wise et al. reported that BPA did not alter the number of astrocytes in the rat prefrontal cortex (53). On the other hand, S100β is known as a neurotrophic factor (54) that regulates neuronal development processes such as neurite outgrowth. Thus, abnormal neuronal differentiation resulting from BPA exposure may be partly due to decreases in S100β. The decrease in β III-tubulin-positive cells may be partly induced by a decreased secretion of S100β.

To avoid the potential adverse effects of BPA, BPF is sometimes used as a substitute. BPF has also been detected in various foods and in the human body. Prenatal exposure to BPF, like BPA, caused several behavioral changes, including increased anxiogenic behaviors and depression in mice (55, 56). The expression of several neuropeptide-related genes has also been shown to be affected by BPF in zebra fish (57). The binding capacity of BPF to ERs was equivalent to that of BPA and BPF-activated ERs. These reports indicate that BPF may disrupt ER-mediated processes, potentially having adverse effects on the brain. In the present study, we did not observe any changes in neural differentiation following BPF treatment. Thus, at least regarding neural differentiation, BPF may have less adverse effects than BPA.

In conclusion, BPA may disrupt the neural differentiation of human-derived NPCs. Such alterations may cause abnormal brain development following gestational exposure.

## Author Contributions

YF and WM conducted the complete experiment and prepared the data and manuscript. WM, TK, and NK had the responsibility for the whole experiment, earned the grant, made the strategy, and prepared the manuscript. YF and WM contributed equally to this work.

## Conflict of Interest Statement

The authors declare that the research was conducted in the absence of any commercial or financial relationships that could be construed as a potential conflict of interest.

## Supplementary Material

Figure S1 mRNA levels of ESR1 and ESR2 in differentiated ReNcell VM cells exposed to BPA, BPF, and E2. Cells were harvested after 3 days exposure to 10^–10^ M of these compounds and extracted total RNA. Real-time quantitative PCR analyses were performed using a primer set for ESR1 or ESR2. Data are represented as mean ± SEM, *n* = 3.

Figure S2 mRNA levels of Rtp1 in differentiated ReNcell VM cells exposed to BPA, BPF, and E2. Cells were harvested after 3 days exposure to 10^–10^ M of these compounds and extracted total RNA. Real-time quantitative PCR analyses were performed using a primer set for Rtp1. Data are represented as mean ± SEM, *n* = 3.

## Abbreviations

BPA, bisphenol A; BPF, bisphenol F; NPCs, neural stem/progenitor cells; DA, dopaminergic, VM, ventral mesencephalon.
