# Supplementary figures and images for: The Effects of Low-Dose Bisphenol A and Bisphenol F on Neural Differentiation of a Fetal Brain-Derived Neural Progenitor Cell Line
Source: Front Endocrinol (Lausanne). 2018 Feb 9;9:24. doi: 10.3389/fendo.2018.00024 (PMC5811521; doi:10.3389/fendo.2018.00024)

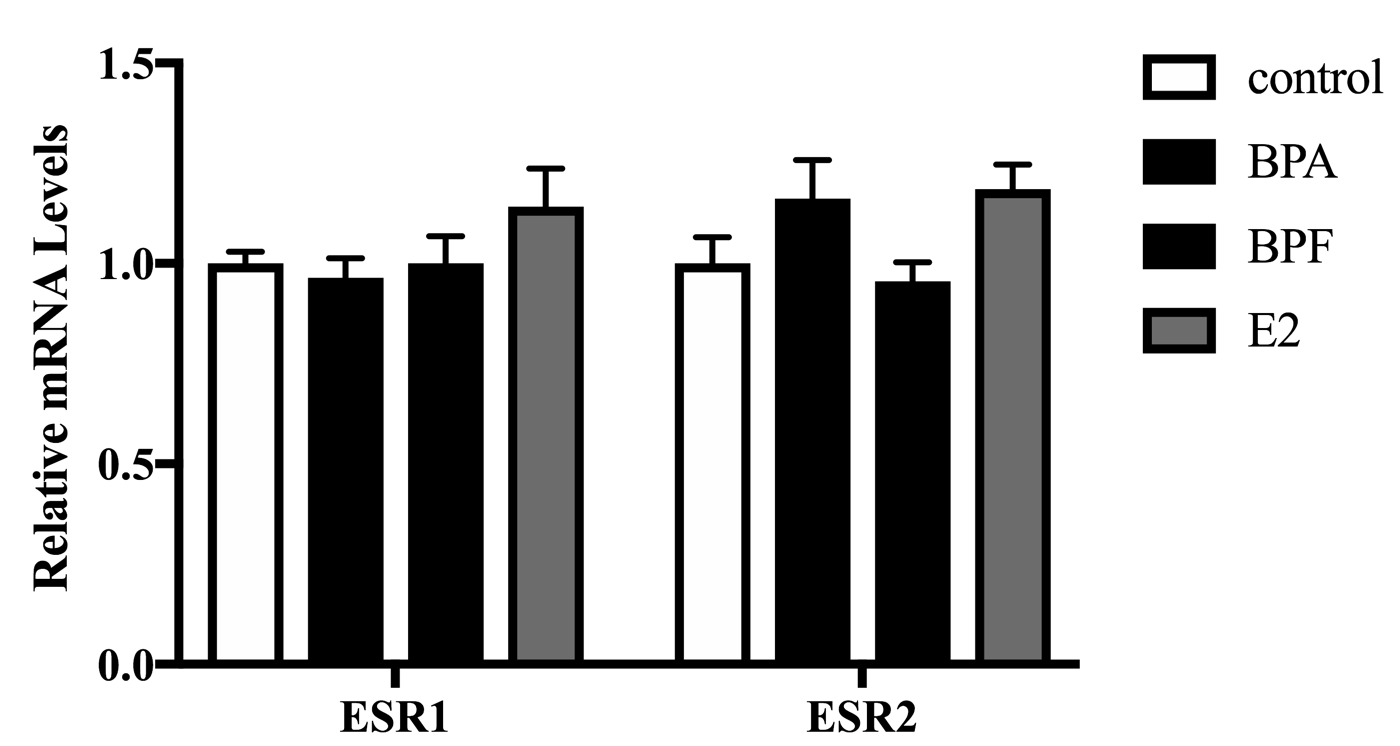

Supplement: Figure S1 — mRNA levels of ESR1 and ESR2 in differentiated ReNcell VM cells exposed to BPA, BPF, and E2. Cells were harvested after 3 days exposure to 10–10 M of these compounds and extracted total RNA. Real-time quantitative PCR analyses were performed using a primer set for ESR1 or ESR2. Data are represented as mean ± SEM, n = 3. [file image_1.tiff]

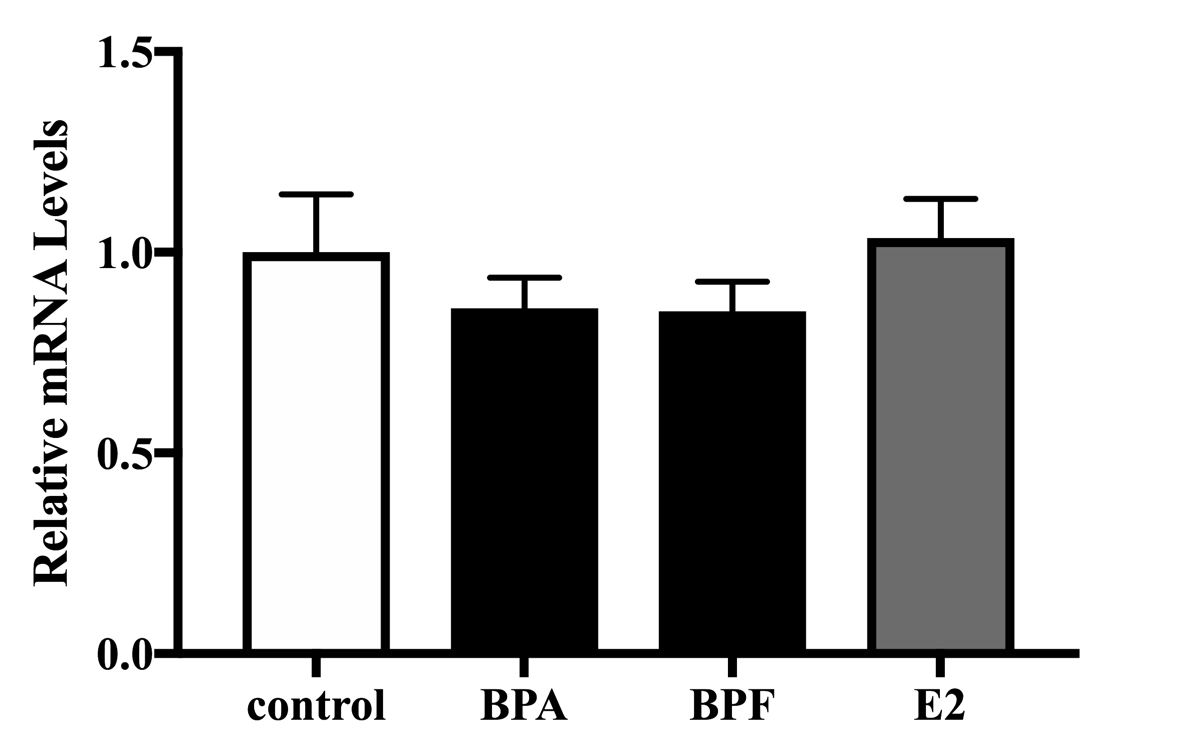

Supplement: Figure S2 — mRNA levels of Rtp1 in differentiated ReNcell VM cells exposed to BPA, BPF, and E2. Cells were harvested after 3 days exposure to 10–10 M of these compounds and extracted total RNA. Real-time quantitative PCR analyses were performed using a primer set for Rtp1. Data are represented as mean ± SEM, n = 3. [file image_2.tiff]
